# Mining HPV Vaccine Knowledge Structures of Young Adults From Reddit Using Distributional Semantics and Pathfinder Networks

**DOI:** 10.1177/1073274819891442

**Published:** 2020-01-08

**Authors:** Muhammad Amith, Trevor Cohen, Rachel Cunningham, Lara S. Savas, Nina Smith, Paula Cuccaro, Efrat Gabay, Julie Boom, Roger Schvaneveldt, Cui Tao

**Affiliations:** 1 School of Biomedical Informatics, The University of Texas Health Science Center at Houston, TX, USA; 2 Biomedical Informatics and Medical Education, University of Washington, Seattle, WA, USA; 3 Texas Children’s Hospital, Houston, TX, USA; 4 School of Public Health, The University of Texas Health Center at Houston, TX, USA; 5 Arizona State University, Tempe, AZ, USA; 6 New Mexico State University, Las Cruces, NM, USA

**Keywords:** distributional semantics, HPV, Reddit, social media, Pathfinder networks, graph theory, health promotion, word embeddings, young adults, knowledge representation, vaccine

## Abstract

The human papillomavirus (HPV) vaccine protects adolescents and young adults from 9 high-risk HPV virus types that cause 90% of cervical and anal cancers and 70% of oropharyngeal cancers. This study extends our previous research analyzing online content concerning the HPV vaccination in social media platforms used by young adults, in which we used Pathfinder network scaling and methods of distributional semantics to characterize differences in knowledge organization reflected in consumer- and expert-generated online content. The current study extends this approach to evaluate HPV vaccine perceptions among young adults who populate Reddit, a major social media platform. We derived Pathfinder networks from estimates of semantic relatedness obtained by learning word embeddings from Reddit posts and compared these to networks derived from human expert estimation of the relationship between key concepts. Results revealed that users of Reddit, predominantly comprising young adults in the vaccine catch up age-group 18 through 26 years of age, perceived the HPV vaccine domain from a virus-framed perspective that could impact their lifestyle choices and that their awareness of the HPV vaccine for cancer prevention is also lacking. Further differences in knowledge structures were elucidated, with implications for future health communication initiatives.

## Introduction

An estimated 1 in 4 people in the United States are currently infected with the human papillomavirus (HPV) and 14 million additional people are infected with HPV each year. While most HPV infections (90%) are cleared within 2 years, persistent infection with high-risk HPV infections causes 90% of cancers of the cervix and anus and 79% of throat cancers. Consequently, Gardasil 9 (HPV vaccine licensed for both males and females)^1^ is recommended for adolescents at 11 to 12 years of age.^2^ In addition to vaccinating younger teenagers, the Centers for Disease Control and Prevention (CDC) recommends vaccinating females from 13 to 26 years old and males from 13 to 21 years old if they have not yet been vaccinated adequately. The recommendation to vaccinate males extends to age 26 if they have compromised immune systems (eg, HIV infection) or are gay, bisexual, transgender, or have sex with other men.^1^ While clinical guidelines encourage vaccination for both males and females, in the United States, young adult women are much more likely than their male counterparts to have been vaccinated.^3-6^ However, the HPV vaccination coverage rate is alarmingly low for both males and females (8.2% and 40.2%, respectively, for young adults from 19 to 26 years old^7^).

In the United States, while parents typically are the decision makers regarding vaccination for minors (up to age 18), young adults make the decision to vaccinate themselves. However, research has shown that parental opinion may still influence vaccine uptake in this population.^8,9^ Studies have also uncovered other patterns surrounding HPV vaccination uptake among US young adults. Health-care access plays a role in vaccine uptake; studies have found that access to free vaccination was strongly predictive of vaccine uptake, cost was a barrier to vaccination, and knowledge of where to receive vaccinations was correlated with a more favorable view of vaccination effectiveness and safety.^8,10-12^ Other studies found that having an established relationship with a primary doctor, having received a non-HPV vaccine, and having received HIV testing were predictive of vaccination, but having health insurance was not always associated with vaccine completion.^4,11,13-15^ Furthermore, being of a racial/ethnic minority, having lower educational attainment, and preferring a language other than English for communicating health information was associated with less vaccination.^10,15,16^ There are also geographic variations in vaccination rates, with areas of more poverty seeing less HPV vaccination.^12,17^ More research is needed about factors that encourage vaccination initiation among US young adults (the first of a series of 2 or 3 vaccinations, depending on patient age and vaccination interval) and series completion because studies suggest that barriers for initiation are likely different than the barriers for completion.^4,12,16-18^

Studies of HPV vaccine acceptability, beliefs, and knowledge among young adults in the United States found low perceived susceptibility to HPV, especially among those in committed relationships.^3,12,19^ Studies also found that while knowledge of HPV infection among young adults has improved over the past decade, misconceptions about vaccine safety and shortcomings in HPV literacy still exist.^5,9,12^ Misunderstandings differ among young adult subpopulations. One study by Klosky et al^20^ on HPV vaccination among young adult cancer survivors found them to be more HPV vaccine naive than the general population despite their experience with cancer. In another study, Vanderpool et al^17^ found that in a rural area with high cancer rates, fatalism about developing cancer predicted nonvaccination. Studies suggest that in the United States, provider education and recommendation of vaccination to young adults could encourage uptake, but research also suggests the need for more culturally tailored messaging, both in delivery and in format.^3,4,10,12,15,20,21^

One potential outlet for HPV vaccination information is through social media. The term “social media” refers to Internet-based platforms that allow users to contribute and share information. The majority of Americans now access social media and young adults are particularly heavy users.^22^ Use of social media to seek health information, facilitate social support, and promote greater psychological well-being is higher among young adults,^23,24^ making it a novel information source to understand personal determinants of health-seeking behaviors. A study of HPV-related posts in a social media found that information and misinformation influence vaccine acceptance.^25^ Another study on HPV vaccination content on social media found both male and female discussants, highlighting how both genders participate in discourse surrounding this topic on social media.^26^

While many studies have analyzed social media using such as word embeddings, we selectively reviewed previous work applying machine learning (ML) methods to vaccine-related social media content. This review was conducted on PubMed in November 2018 using the following search query: “social media AND (“distributional semantics” OR “machine learning”) AND “vaccine” and sorted by best match. Most research within this area focused on classification-related tasks (sentiment, topics, etc) using various ML approaches and using Twitter as a data source.^25,27-34^ Some, like Pananos and others^35^ and Tangherlini and colleagues,^36^ used Google searches and website blogs, respectively. The former relied on a mathematical model that looked at near elimination of a disease and measles mumps and rubella (MMR) vaccine high uptake as signals for impending reduced vaccination and disease outbreak. Several studies specifically examined the HPV-related domain.^25,28-30,32,34^

The aforementioned studies focused on ML methods to classify information, yet this is only useful if categories are known beforehand. Text categorization of this nature can determine whether or not a previously identified concept has been mentioned or not, but this doesn’t provide any information about how concepts might relate to one another in the text or in the mind of its author. In studies that examined sentiment and emotion in text, there is nuance in bifurcated positive and negative emotion classification. If we were to show the association of data, or the structure of knowledge, more meaningful representation of the data would evince expressive information that can be utilized by researchers and experts. Our work makes use of the combination of distributional semantics and network graphs that can elicit interpretable representations of conceptual relationships from large amounts of unstructured text. Instead of Twitter, we focus on the Reddit social media platform, evoking structures of knowledge from 2 different populations—young adults and health experts. To the best of our knowledge, this is the first study that examines at Reddit on consumer vaccine research.

Social media sites’ data are accessible to evaluators and researchers and can be used to access first-hand, real-time information about experiences and outcomes of patients with cancer.^37^ Social media platforms differ particularly in the demographics of their users.^22^ Reddit is a popular social media platform whose users are mostly in the United States (54%) and younger than 35 years of age (87%).^38^ Reddit is currently the fifth most accessed website in the United States and the seventh most accessed website in the world.^39^ Referring to itself as “The Frontpage of the Internet,” Reddit is the first source of news for a sizable number of its users.^40^ Signing up for a Reddit account is free and allows the user to participate in subreddit communities that share and discuss content on a common interest. Reddit account holders can post and “vote” on content. They “upvote” posts that they find interesting and “downvote” posts that they do not like. A post with more “upvotes” rises to the top of a subreddit home page. As such, the Reddit platform is a real-time proxy for trending interests and values among its users.^38^ Data from Reddit have been used by researchers to examine health behaviors,^41^ obtain information about patient experiences,^42^ track patient outcomes,^43^ and identify common health information needs, perceptions, concerns, and health beliefs.^43-47^ Our study used Reddit to examine the perceptions, concerns, and beliefs of Reddit users as they pertain to HPV vaccination. Findings from our study will increase understanding of factors influencing young adults’ decision-making regarding obtaining the HPV vaccination and will help guide future interventions targeting HPV vaccination uptake in this population. Ultimately, methods used and findings from this work will inform future approaches to conducting Internet-based formative work and e-behavioral intervention research targeting online groups, such as young adults.

## Distributional Semantics

Distributional semantics is predicated on the notion that words that appear in similar contexts across large bodies of free text (corpora) may be semantically related to each other.^48^ Take, for example, the words “cancer” and “neoplasm” that appear frequently in a hypothetical corpus with the word “cervical.” The 2 terms mentioned may be related on account of their frequent presence in similar contexts. Since the 1990s, several distributional semantic approaches have been introduced that derive geometric representations of terms (word vectors or word embeddings) from their occurrence across large text corpora.^49,50^ Often referred to as word space (or semantic space) models, these models have been widely used because (1) they represent words and their meanings as a geometric representation that is amendable to further computation, (2) they utilize a data-driven approach to understand meaning from context without any prior linguistic or semantic knowledge, (3) they permit application of simple vector operations to extrapolate information and generate representations of larger units of text, and (4) estimates of relatedness between words can be derived from their vector representations.

Latent semantic analysis is a seminal word space model that generates a reduced dimensional approximation of a statistically weighted term-by-document matrix and estimates relatedness between words from the distance between the resulting word vector representations.^51^ Hyperspace Analogue to Language (HAL)^52^ is a related approach where, instead of a word-by-document matrix, each row (word) in a term-by-term co-occurrence matrix captures its frequency of co-occurrence with other words in a sliding window moved through the text. The Skip-Gram model^53^ is another window-based approach that uses artificial neural networks to predict terms that occur in context with a target term and derives estimates of semantic relatedness from the neural network weights for each term in a trained model.

### Random Indexing

In this study, Random Indexing (RI),^54^ a stochastically implemented distributional semantic method based on the notion of sparse distributed memory,^55^ and Reflective Random Indexing (RRI),^56^ an extension of RI that aims to enhance its ability to recover meaningful implicit associations between terms from a corpus,^57^ were used. One benefit of RI is the reduced dimensionality of the vectors which are obtained without the need to represent a full term-by-context matrix explicitly, enhancing scalability. Another unique benefit is that it is not reliant on a specific data structure (ie, term-by-document or term-by-term matrix), which means it is adaptable to any word context scheme.

With RI, each unique document in a target corpus is denoted by a stochastically generated random index vector with a predefined dimensionality and seed length. The vector is initialized as a vector of zeros, but a small number of elements (on the order of 10) are randomly assigned to “1” or “−1.” In addition to the random index vector, each unique term in the corpus is assigned a semantic vector. This semantic vector represents semantic information derived from random index vectors representing the contexts in which a term occurs.

**Figure 1.**
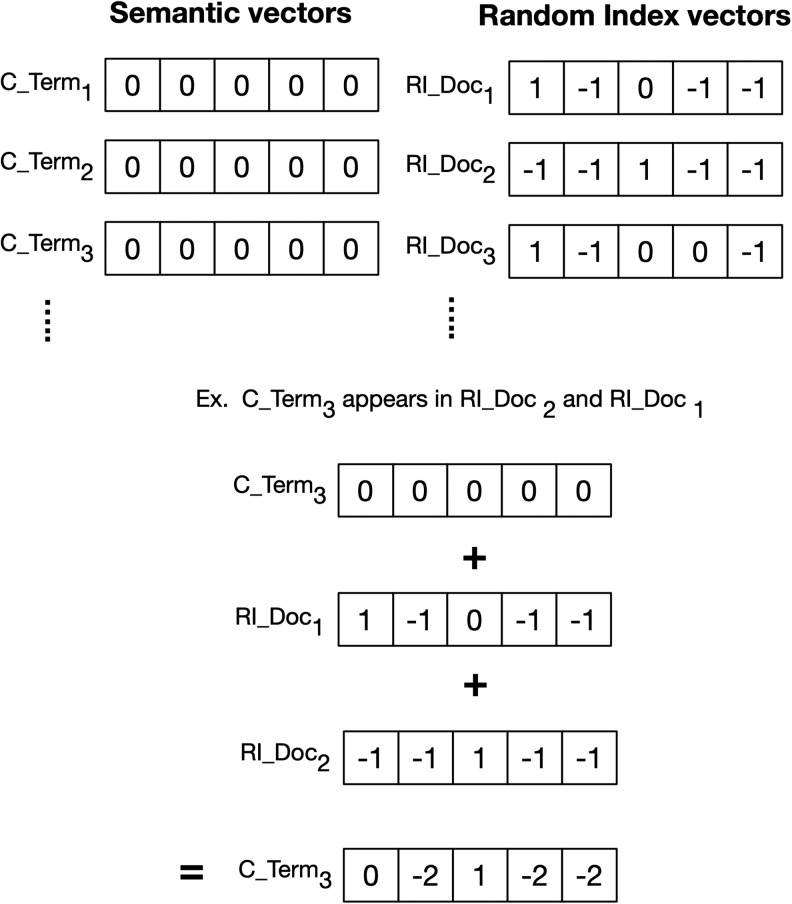
Simple example of Random Indexing (RI) allocation of context vectors for terms. For RI, the random index vectors represent document occurrence.

The training of the semantic vectors is accomplished by traversing the documents in a corpus. In Figure 1, each term found in a document will have the document’s random index vector added to the term’s semantic vector. The net result provides us with scalable low-dimensional vectors for terms.^54^ To add one qualification to the summary, instead of random index vectors for documents, we can also alternatively assign random index vectors to terms which would permit us to apply RI to approximate sliding window-based approaches such as HAL.^54,58^ Directional models further extends the sliding window approach through permutation of the random vectors of terms that appear before or after certain terms, such that terms are encoded differently depending on their orientation to a focus term in a sliding window.^59^

**Figure 2.**
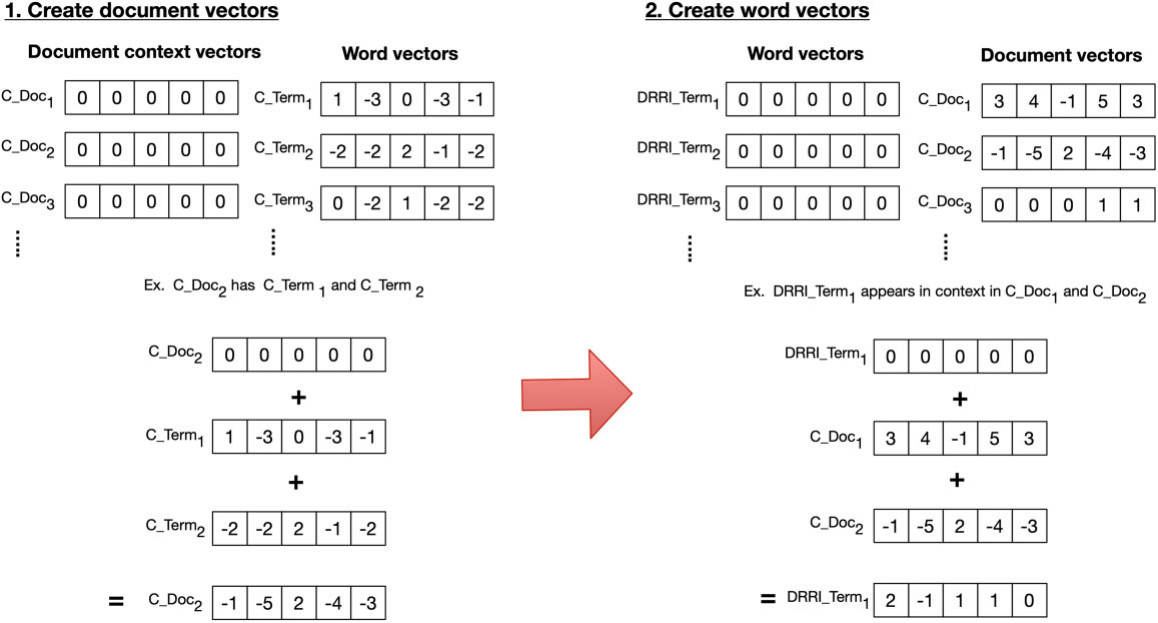
Simple example showing Document-Based Reflective Random Indexing (RI) steps after preforming RI (refer to Figure 1).

**Figure 3.**
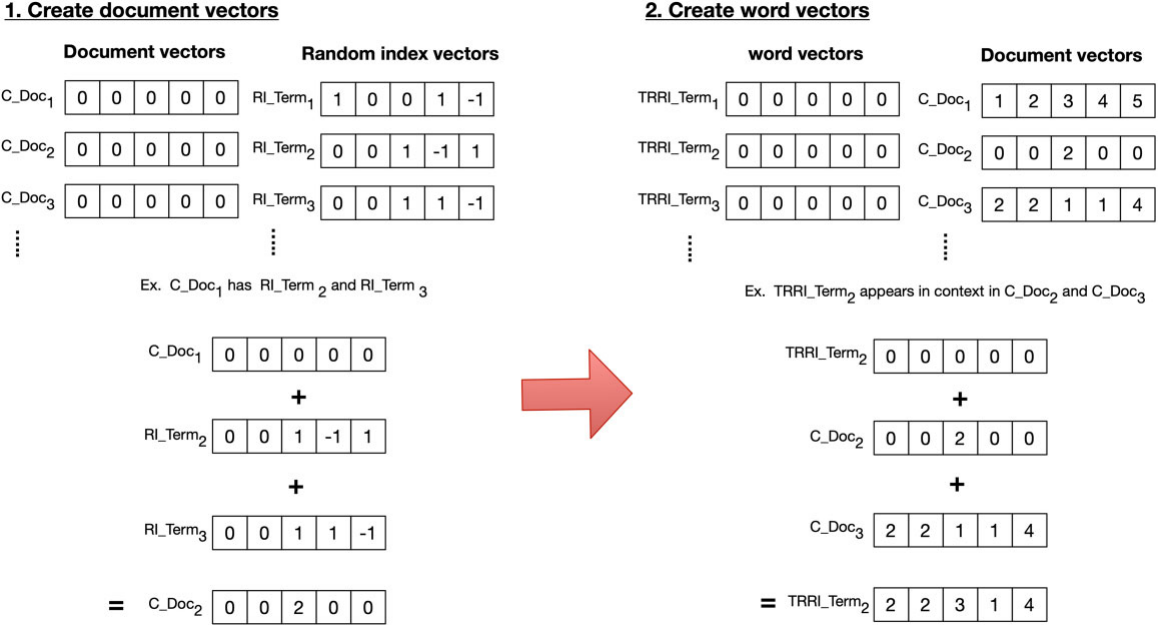
Simple example showing the Term-Based Reflective Random Indexing method.

Reflective Random Indexing involves additional steps depending on the type—Document-Based Reflective Random Indexing (DRRI) or Term-Based Reflective Random Indexing. For DRRI, another allocation step occurs after the standard RI method where another set of document vectors is derived from the semantic vectors previously produced in standard RI by adding together the vectors for terms that occur in documents (often with statistical weighting). This in turn can be used to generate another set of semantic vectors, as shown in Figure 2. With each term that appears in a document, the term’s previously trained word vector is added to the document vector (step 1 in Figure 2). Then, from the resulting document vectors, the same process is repeated in RI using the newly created document vectors (step 2 in Figure 2). Term-based RRI, unlike DRRI, does not initially start with the standard RI method. Instead, illustrated in Figure 3, each unique term in the corpus is assigned a random index vector and terms that appear within specific documents have their random index vector added to a document vector for the document. With the resulting document vectors, each unique term is assigned a new word vector, to which the document vector for each document the term appears in is added—creating a semantic term vector. Ultimately, the RI and RRI methods both result in reduced dimensional word space vectors that can be used to calculate cosine similarity between each term vector and to derive proximity data that can produce Pathfinder networks (PFNETs). Implementation of RI and RRI is available in the open source Semantic Vectors package.^60^

### Pathfinder Networks

Pathfinder networks were introduced by Roger Schvaneveldt and his colleagues in the 1980s.^61^ Pathfinder network scaling—the method used to derive PFNETS—is a psychometric method that harnesses graph theory approaches to identify salient links between concepts in order to represent mental models or knowledge structures of groups or individuals. The underlying idea is that the links preserved between network nodes representing concepts reflect the cognitive and psychological structures representing these concepts in the minds of individuals. Typically, these network links are based on individual judgments of similarity between concepts (eg, semantic similarity data), but networks have also been obtained from estimates of semantic relatedness derived using methods of distributional semantics. The core network structure of the PFNETs is a minimal weighted subgraph (sometimes a minimal spanning tree depending on parameters) of a network derived from an algorithm to filter less essential links. It has been shown that these minimal subgraphs express the core relationships of the network that reflect memory and knowledge structures.^62^

The algorithm for PFNET transforms a network graph with links that represent distances among the various nodes governed by 2 parameters—*r* and *q* noted as PFNET(*r*, *q*). The *q* parameter constrains the number of steps an alternative link (ie, number of links) may have. This allows control over the density of the graph by regulating the number of links in the PFNET. From a representational standpoint, the *q* value also modulates the number of links for psychological interpretation and expressiveness. Often the parameter of *q* = *n* − 1 is selected, imposing no limit on the number of links considered. The *r* parameter governs the distance metric used to determine the length of each path. This is important because the pruning aspect of the PFNET uses the weights of individual links in a network path. This aspect is predicated on the Minkowski distance measurement where if the *r* parameter is ∞, we take the maximum weight of links (*w*_1_, *w*_2_,…, *w*_k_) to determine the length of a path comprising these links (Equation 1), or if *r* = 1, the weights of the links is added to determine filtering (Equation 2) for their path. Other values for *r* may be substituted which would affect the sum of the link’s weights (Refer to Equations 1 and 2). Using the *r* parameter, the triangular inequality heuristic is applied to specify removal of a network link, toward a sparser graph. For a more detailed discussion on the parameters, refer to the study by Schvaneveldt and associates’ thorough introduction on PFNETs.^61^

1 $$w\left(P\right)=\underset{r\to \infty }{lim{\left[w_{x}^{r}+w_{y}^{r}\right]}^{1/r}}=\text{max}\left(w_{x},w_{y}\right).$$

2 $$w\left(P\right)={\left[\overset{k}{\underset{i=1}{\sum }}w_{i}^{r}\right]}^{1/r},\;r\geq 1,\;w_{1}\geq 0.$$

**Figure 4.**
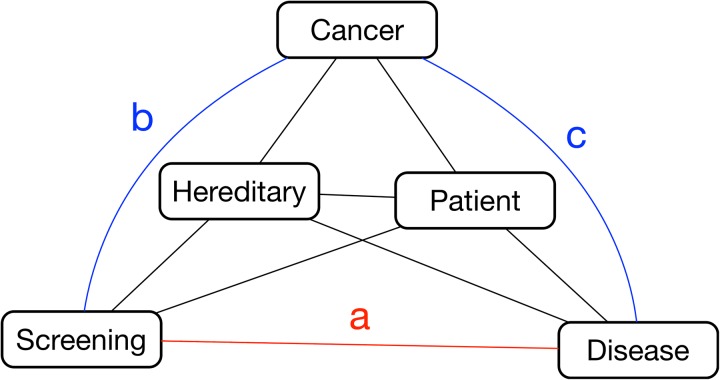
Hypothetical network graph with links a, b, and c for assessing filtering.

To illustrate the basis of PFNET, given a hypothetical network graph in Figure 4, every network link that corresponds with a target link is evaluated to assess triangular inequality. The target link is *a* and links *b* and *c* are assessed for the heuristic. If *r* = 1, *b* and *c*’s link weights are added, and if the added weight is less than the link weight of *a*, link *a* is pruned out, otherwise it remains. If *r* = ∞, the maximum weight of *b* and *c* is compared to the weight of link *a*. Similarly, if it is less, link *a* is pruned, otherwise it remains. This continues until every possible triangular link in the network graph with link *a* has been evaluated up to the number of links defined by the value of *q*.

Overall, these parameters have the effect of creating slightly different networks depending on the value of the parameters. Typically, when *r* = ∞ and *q* = *n* – 1, where *n* is the number of nodes, the resulting network is a minimal network with nonsalient links removed. In most cases, these sparser networks capture the important structures that could reflect the knowledge organization of individuals.^62^

## Research Objective

In a previously published study,^63^ we explored the use of RI and RRI as a basis for PFNETs revealing knowledge structures of health consumers and health experts on the topic of vaccines, where the weights of the paths between concepts in the PFNETs were obtained from the semantic distance derived from word space models of consumer- and expert-authored content. The PFNETs revealed significant differences in perspectives relating to the vaccine domain where consumer knowledge structures centered around controversial notions about vaccines, compared to experts who structured their knowledge in accordance with scientific consensus on vaccines.^63^ Motivated by our results in that study, we aimed to extend the methodology to a corpus of Reddit messages that involve the HPV vaccine. The aim of this study was to derive and analyze consumers’ knowledge and perceptions on vaccination (particularly HPV vaccination in our case) from topic-related submissions posted on Reddit, a social media platform. The results were expected to show how young adults who utilize Reddit conceive of the HPV vaccine and its related concepts. Understanding preconceptions of this group could inform health-care professionals to better engage target vaccine audience users on social media platforms such as Reddit.

The following study utilized PFNETs to interpret the set of relationships existing between extracted keywords from submissions posted on Reddit over a duration of 10 years (2007-2017). Unlike our previous work, this study addresses a specific type of vaccine, namely the HPV vaccine, and how a young adult demographic, a demographic that is at risk for HPV,^64,65^ conceives of this domain. We also derived a PFNET from expert ratings of concepts instead of curating a corpus of expert-authored content. We enlisted the help of participating health experts (eg, pediatricians, health communication experts, behavioral scientists, and epidemiologists focused on HPV vaccination research, etc, from the Texas Medical Center) to provide their ratings for pairs of HPV vaccine concepts to produce an expert knowledge structure of the HPV vaccine domain as a PFNET. We proposed, by using the generated knowledge structures of PFNETs for experts (ratings based) and Reddit community members (distributional semantics based), to understand:

- R^1^: What are the principal notions about the HPV vaccine domain in the Reddit community?
- R^2^: What are differences in HPV and HPV vaccination knowledge and perceptions between Reddit users and HPV vaccine experts?

## Method

### Seed Terms

To derive PFNET for entities in the domain knowledge structure of Reddit contributors, a list of concepts that would serve as the nodes was required. Demographically, Reddit users are a distinct user group from those of other social media sites. Most Reddit users reside in the United States and are younger than 35 years of age.^38^ With this in mind, a list of concepts from a review of the literature on HPV vaccination and young adults was compiled. PubMed was searched for articles with keywords “HPV” and “vaccination.” Given the Reddit user demographics, articles were chosen if they described data from the United States and described the issue from the perspective of those who were considering vaccination for themselves (students, adolescents, young adults, and not parents). After identifying an article of interest from this list, articles PubMed had suggested as “articles like this one” and “articles that cited this” were also examined. Concepts, based on the ConceptNet knowledge base^66^ (a semantic knowledge graph of “commonsense” information), were extracted based on repetition and similarities across articles. Concept identification continued until themes appeared to be saturated (n = 10 articles, of which 5 were literature reviews). Researchers then met with medical and public health content experts in the Texas Children’s Hospital and the University of Texas Health Science Campus to prepare a final list of terms for the analysis. As we will explain later, the concept pap screen was excluded from analysis due to its absence from the Reddit corpus. The final list of 23 concepts (including pap screen) was:

**Table:** 

Big pharma	HPV vaccine	Recommendation
Cancer	Inaccessible	Risk
Death	Information	Scientific evidence
Doctor	Myth	Side effects
Family	Pain	Trust
Genital warts	Pap screen	Unnecessary
Health	Prevention	Unsafe
HPV	Promiscuity	

### Reddit Corpus Processing

The Reddit corpus for HPV-related content was derived from a data set from Pushshift.io.^67^ Submissions (topic starters) and comments (responses to the topic) that contained the case-insensitive expressions of “hpv,” “papillomavirus,” “cervarix,” or “gardasil” were extracted and stored in a PostgreSQL database. The entire subset of HPV-related content was then exported to plain text files. The entire Reddit corpus was also processed with word2phrase^68^ to identify and concatenate multiworded tokens (ie, “HPV vaccine,” ‘Rapa Nui,” etc). In total, the number of documents from the corpus was 88 836.

### Human Papillomavirus Vaccine Rating for Expert PFNET

**Figure 5.**
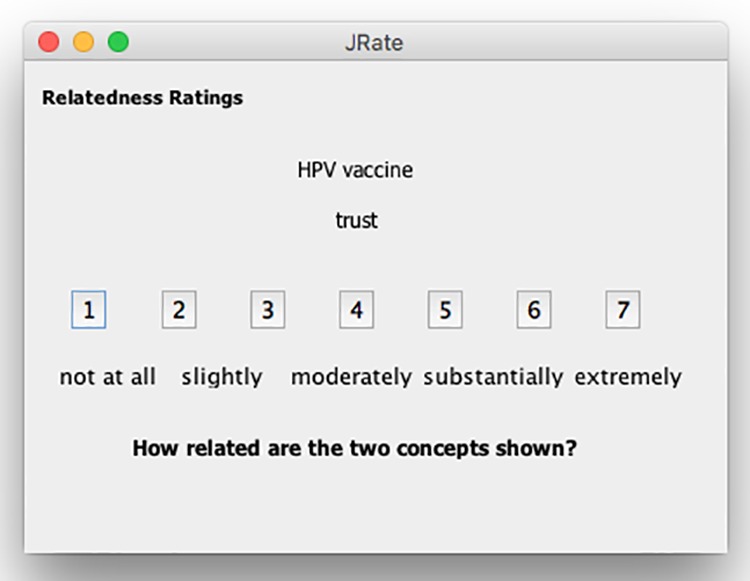
JRate screenshot with sample concepts.

Four HPV vaccine experts who were either an MD and/or held an MPH in public health volunteered to rate the association of the seed terms. Using JRate^69^ facilitated the collection of pair-wise relatedness ratings. The seed term concepts were presented individually with a prompt asking the rater to determine how related the 2 concepts are (ie, 1 for not at all related, 7 for extremely related). Figure 5 displays the interface of JRate that raters viewed for pairs of concepts. The resulting data were exported to a Pathfinder proximity data file.

### Generating Proximity Data for Reddit PFNET

The Semantic Vectors software package^70^ (prerelease version 5.9) was used to derive the proximity data from the seed terms with word space models. Then several word space models were created—RI and its variant models and a neural embedding model derived using the Skip-Gram with negative-sampling algorithm.^53^ For sliding window models, we utilized a contextual window size of 10 (radius = 10) to capture both synonymous and associative relationships.^71^

- Term by document RI
- Term-based RRI
- Document-based RRI
- Sliding window (window size of 10; RI variant)
- Directional window (window size of 10; RI variant)
- Skip-Gram neural embedding (window size of 10 and 9 training cycles; 200 dimensional size)

For each of the models, we incorporated a stop word list from Cornell University researchers’ SMART information retrieval system.^72^ Inverse document frequency weighting was utilized for the RRI models.

**Figure 6.**
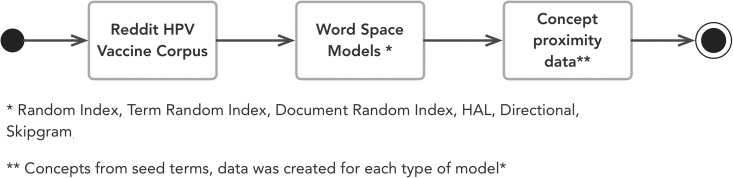
Summary of the process to build word space models. *Random index, term random index, document random index, Hyperspace Analogue to Language, directional, Skip-Gram. **Concepts from seed terms, data were created for each type of model.

After the 6 abovementioned word space models were built, the vector space with each seed term (concept) was extracted from each model and the cosine distance between each concept calculated. The data were formatted in a proximity data file (similar to the JRate export). The exported proximity data were analyzed and used to generate the PFNET (discussed in Results) through JPathfinder,^73^ a freely available software tool to explore and visualize PFNETs. Figure 6 summarizes the process of creating the proximity data for the seed concepts. Java software code for preprocessing of the corpus and the creation of the proximity data are available at http://bit.ly/2sOBZkc.

## Results

### Coherence Data

The coherence score for each of the models produced was measured (see Table 1). The coherence score assesses the transitivity of the pair-wise associations for an individual proximity word model. A low coherence score indicates completely random associations, whereas a high rating suggests meaningful associations. With the expert ratings from JRate, all 4 of the individuals’ ratings were merged using the built-in feature provided by JPathfinder. A proximity word model for mean and median ratings was produced and coherence score for both derived. The coherence scores for the various Reddit proximity word models were also derived.

**Table 1.** Coherence Scores for Proximity Word Models.

Model	Coherence
Expert rating (mean)	0.726
Expert rating (median)	0.715
Directional RI	0.723
Document RRI	0.719
Sliding window RI	0.688
Term RRI	0.643
Skip-Gram	0.508
RI	0.488

Abbreviations: RI, Random Indexing; RRI, Reflective Random Indexing.

**Figure 7.**
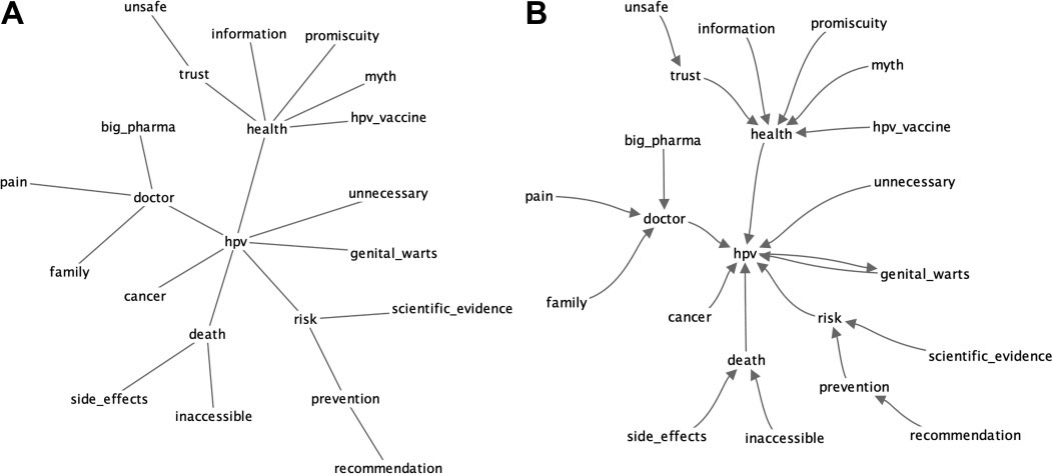
Network representation of Reddit knowledge structure, derived from Reddit corpus. A, PFNET of Reddit knowledge structure, derived from Reddit HPV-centric corpus. B, Nearest neighbor network of Reddit knowledge structure, derived from Reddit HPV-centric corpus.

**Figure 8.**
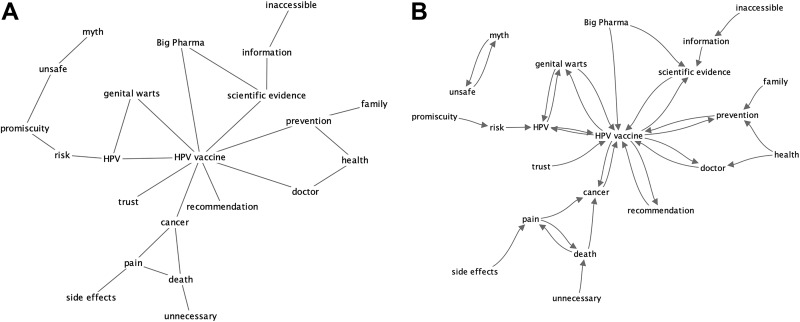
Network representation of experts’ knowledge structure from Jrate. A, PFNET of experts’ knowledge structures from JRate. B, Nearest neighbor network of expert’s knowledge structure.

Comparing all of the Reddit proximity word models, the directional (with RI) model yielded the highest coherence (0.723) compared to the rest, with document RRI model with the second highest rating (0.719). The mean and median version of expert rating produced similar results (0.726 and 0.715). From Figures 7 and 8, the directional and mean proximity for the expert rating were compared and we generated the visualization and its accompanying meta-data.

### Pathfinder Network Data

Network visualizations and meta-data were generated from JPathfinder. Figure 7A and Figure 8A show the PFNETs representing the knowledge structures for Reddit users and experts. Supplementing the PFNETs are visualizations of the nearest neighbor network (directed graph) to reveal subgraphs (Figures 7B and Figure 8B). Essentially, the nodes for a nearest neighbor network point to the closest (ie, most similar) node of the network. The links of a nearest neighbor network will appear on PFNETs, although PFNETs may have additional links. In our previous study, nearest neighbor network representations highlighted distinct subgroups of concepts.^63^ Table 2 details some important properties of the 2 networks. The *pap screen* concept did not appear in the Reddit network due to not appearing in the Reddit corpus. Hence, we removed that concept from the expert network and recomputed the network to provide comparable networks with equal number of nodes. The Reddit PFNET exhibited 22 nodes and 21 links connecting the nodes, while the expert PFNET exhibited 22 nodes and 25 links within its network.

**Table 2.** Comparison of Pathfinder Network Data.

	Reddit	Expert
Number of links	21	25
Number of nodes	22	22
Central concept		
(Eccentricity)	HPV (3)	HPV (4)
Concept w/		
Maximum degree	HPV (7)	HPV vaccine (9)
Shared links	2	

Abbreviation: HPV, human papillomavirus.

Calculating eccentricity value (ie, of the number of links between a node from the farthest other node) for a network representation can indicate a node that is central within an entire network: the node with minimum eccentricity. A central concept in a network structure could indicate the arrangement of knowledge based on a particular core concept, and in the case of representing knowledge, it could indicate the important unifying belief of the network. Aside from eccentricity, the maximum of number of links to a concept (maximum degree) can also emphasize an important concept. For example, for the Reddit PFNET, the central concept, based on eccentricity calculation, was *hpv*, while the expert PFNET was *hpv*. The Reddit PFNET concept of *hpv* has the maximum number of links to it, and *hpv vaccine* has the maximum number of links within the expert PFNET. Finally, between PFNETs of Reddit and expert, the only two shared links are *risk* ↔ *hpv* and *hpv* ↔ *genital warts*.

Figure 8B exhibits a group of isolated nodes. These are linked concepts that are separate from a larger network, often specifying some disconnect with the meaning or the expression of the larger network. For Figure 8B, *unsafe* and *myth* are a set of concepts, disconnected from the larger expert network. No isolated subnetworks exist for the Reddit network (Figure 7B). These isolated nodes are likely to have no association with the larger network due to having no or very semantic similarity with other interconnected Reddit concepts. In other words, these nodes have little meaning with the other concepts from the Reddit corpus.

### Pair-Wise Similarity Data

Tables 3 and 4 presents pair-wise similarities of the concepts for Reddit community and the experts. For each of the edges, the similarity score was calculated to indicate how closely associated each pair of concepts were to one another (ie, a shorter distance representing close association of the concepts). In Table 3 of the Reddit model (directional), the similarity association was derived from a cosine calculation. In Table 4 of the expert model (JRate), the distance was computed by JPathfinder from the Likert ratings.

**Table 3.** Reddit Pair-Wise Similarity.^a^

		Similarity
genital_warts	hpv	0.823
cancer	hpv	0.798
hpv	risk	0.764
doctor	hpv	0.669
health	hpv	0.628
doctor	family	0.598
health	trust	0.589
health	information	0.536
hpv	unnecessary	0.528
prevention	risk	0.495
death	hpv	0.494
doctor	pain	0.487
death	side_effects	0.464
health	myth	0.398
trust	unsafe	0.396
big_pharma	doctor	0.370
health	promiscuity	0.351
risk	scientific_evidence	0.304
health	hpv_vaccine	0.254
death	inaccessible	0.243
prevention	recommendation	0.233

^a^ Green highlights show high similarity score (μ + 1σ) and red highlights low similarity (μ − 1σ). Similarity scores ranged from 0 to 1, with 1 indicating exact similarity association and 0 for no similarity.

**Table 4.** Expert Pair-Wise Similarity Ratings from JRate.^a^

		Similarity
cancer	HPV vaccine	7
doctor	HPV vaccine	7
genital warts	HPV	7
genital warts	HPV vaccine	7
HPV	HPV vaccine	7
HPV vaccine	prevention	7
HPV vaccine	recommendation	7
HPV vaccine	scientific evidence	7
cancer	death	6.75
cancer	pain	6.75
death	pain	6.75
doctor	health	6.75
health	prevention	6.75
HPV vaccine	trust	6.75
information	scientific evidence	6.75
family	prevention	6.25
HPV	risk	6
promiscuity	risk	5.75
myth	unsafe	5.5
pain	side effects	5.5
promiscuity	unsafe	4.5
Big Pharma	HPV vaccine	4.25
Big Pharma	scientific evidence	4.25
inaccessible	information	3.75
death	unnecessary	3

^a^ Green highlights show high similarity score (top 20%) and red highlights low similarity (bottom 20%). Similarity ratings ranged between 1 to 7, with 7 indicating extremely high relatedness and 1 for no related similarity.

The most and least close association of concept pairs among the Reddit model had similarity and dissimilarity distances beyond 1 standard deviation among all pairwise associations. The pair-wise similarities of *genital warts* ↔ *cancer*, *cancer* ↔ *hpv*, and *risk* ↔ *hpv* are highly associated concepts that are 1 standard deviation beyond the average similarity (μ + 1σ). Concept pairs such as *risk* ↔ *scientific evidence*, *health* ↔ *hpv vaccine*, *death* ↔ *inaccessible*, and *prevention* ↔ *recommendation* (weakly associated pairings) are 1 standard deviation below the average similarity(μ − 1σ).

Among the experts (Table 4), the concept pairs that are closely associated were highlighted in green (similarity value of 7). There are also weak pair-wise associations for the Reddit and expert model. *hpv vaccine* ↔ *health*, *inaccessible* ↔ *health*, *recommendation* ↔ *hpv*, and *scientific evidence* ↔ *death* has indistinct associative pairings among the Reddit pairings. With the expert, the concept pairs with weak associations are highlighted in red. These “weaker” pairs are also 1 standard deviation below the average (μ − 1σ).

## Discussion

From the various data generated from word space models of the Reddit corpus and the Likert ratings for the pair-wise comparison of terms, we discovered discrepancies in the concept similarity and network data that may have implications for future research and communication efforts.

R1: What are the principal notions about the HPV vaccine domain for the Reddit community?

As shown in Table 2, concepts’ maximum number of links (maximum degree) and low eccentricity values were viewed as primary concepts that expressed the theme or idea of the network. For Reddit PFNET, *hpv* is the primary concept, which is different from Expert PFNET where *hpv* and *hpv vaccine* are the primary concepts. Of interest, the seed terms were intended to be related to the *hpv vaccine*, so a Reddit PFNET centered on *hpv* illustrates how Reddit users implicitly arrange their knowledge around virus- rather than vaccine-related topics. In contrast, HPV vaccine experts abstracted their knowledge using professional understanding of the HPV vaccine and HPV. This is further supported when observing the placement of *hpv vaccine* in relation to other concepts from the Reddit PFNET. *hpv vaccine* has one link to the network of the Reddit PFNET. As we had noted earlier, this link is one of the weak associations from that network.

Furthermore, *hpv* and *genital warts* has the highest similarity (0.823). *risk* is another concept with high similarity to *hpv*, and *health* (0.628) has a moderate association with *hpv*. When exploring the nearest neighboring concepts other than the seed concepts (see Appendix Table A1), the concept *risk* relates to concepts such as *contracting*, *transmission*, *infection*, and so on. *health* and *promiscuity* has a weak association (0.351), but the concept *sexual* is one of its nearest neighboring concept outside of the seed concepts. Overall, the PFNET for Reddit expresses young people’s domain understanding from the perspective of STD transmission and the impact of contracting this virus.

R2: What are some differences in knowledge structures between Reddit users and HPV vaccine experts?

Aside from the salient concepts, comparing the associations between the various concepts in the expert PFNET and the Reddit PFNET revealed some additional understanding of young people’s perception of the HPV vaccine domain.

The expert PFNET has numerous concept associations that are highly related, yet many of them do not coincide with the Reddit PFNET. These concepts that are strongly associated are highlighted in green on Table 4.

With the exception of *hpv* ↔ *genital warts* and *hpv* ↔ *risk*, none of these pair-wise associations coincides with the Reddit PFNET. We assert that professional knowledge structures concerning the HPV vaccine are notions that are generally not known or not thought of by young people, especially the association of *hpv vaccine* ↔ *cancer*, which is an important relation in this domain. However, *hpv* ↔ *genital warts* appears to be a common association with this specific population, perhaps because of its important impact on young people’s lifestyle choices. The same can be said for *hpv* ↔ *risk*, which is also another shared pair-wise association. While *hpv vaccine* ↔ *cancer* is associated closely in the expert PFNET structure, we find that *hpv* ↔ *cancer* is highly associated (0.798), indicating some evidence of HPV cancer awareness with this population.

The pap screen concept is absent in the Reddit PFNET structure, as it has no relation/co-occurrence with the other concepts in the word space models for Reddit. Pap screening is an important preventive measure for HPV among females, yet this concept does not appear to be reflected or understood to be important by the younger and predominantly male Reddit population in the context of HPV.

### Implications of the Study

Lack of knowledge and misinformation about HPV and the HPV vaccine are common.^64,74,75^ Moreover, health-care providers often struggle to effectively educate individuals about HPV-related diseases and vaccine.^74,75^ Social media platforms are widely utilized among the younger population and serve as an informational tool for this group. Through the availability of many platforms and their APIs, social media data provide researchers a storehouse of behavioral data collected from a large population.^76^ If experts want to directly communicate and understand this population, researchers need to better wield these platforms for maximum benefit and know the users’ knowledge, attitudes, and beliefs on a specific health topic to refine communication efforts.

One overarching theme that emerged from this study method was an important difference between the young adult population that use Reddit and the HPV vaccine experts. In our examination of Reddit data, we identified common themes including a preoccupation with virus transmission and risk versus awareness of the HPV vaccine. Young adults have high rates of HPV infection.^65,77^ In particular, young adults are also a population who engage in high risk behavior,^78^ which makes them susceptible to HPV transmission. Currently, there is no screening test available for HPV for men (CDC^79^), who may be asymptotic carriers and likely to have a higher number of sexual partnerships.

Another finding is that of lack of knowledge about the HPV vaccine among young adults, possibly due to lack of informational interventions to raise awareness. While experts associate HPV vaccine with cancer and prevention, in our Reddit PFNET, these associations do not emerge. Pap screening was also missing from the Reddit PFNET, possibly because of the predominantly male user population with low knowledge of pap screening to detect cervical cancer or precancerous lesions caused by HPV. To date, few cancer prevention interventions target young adults.^80^ Also, vaccination rates for the HPV vaccine are not meeting the targeted 80%, as HPV vaccination coverage of females and males is at 43%. The HPV vaccine is best administered between the ages of 11 to 25 to attain the benefit of its immunity against the HPV viruses that lead to adulthood cancers, such as cervical cancer and head and neck cancer. While there is strong evidence from our study to support our claims concerning the lack of HPV vaccine awareness, there is awareness for HPV-related cancers and the risks they pose.

This study’s method to mine large corpora on social media to assess consumers’ knowledge of a health-related topic provides a new approach to conducting formative work. What individuals express on social media platforms may be examined qualitatively and quantitatively to identify themes related to how a subgroup may perceive or understand health recommendations, including vaccination. This is particularly helpful for health communication and public health researchers planning e-health interventions targeting online users in specific social media domains. Our approach not only brings to light knowledge structures of a population around certain concepts but also provides insight regarding how these concepts are linked to one another.

Findings from this work indicate HPV vaccination messaging for young adults should focus on genital warts and prevention of HPV infection, followed by education on HPV vaccination for cancer prevention. While cancer prevention may be more acceptable and salient for parents considering the HPV vaccine,^81,82^ the risk of sexually transmitted diseases and their prevention appeared to resonate more with the sexually active young adults. Future studies are needed to confirm these findings, particularly among young adult non-Reddit users, such as young women and those with less educational attainment.

## Conclusion

In this article, we utilized distributional semantics and PFNETs to understand the knowledge structures of Reddit users, reported to be young adults. This enabled creation of PFNETs to discover how closely related certain concepts within the HPV vaccine domain were and to compare them with PFNETs generated from HPV vaccine experts’ ratings. Results show Reddit users do not conceptualize HPV vaccine the same way as experts and that they are mostly concerned with the immediate consequences of the virus itself. Our results have implications for public health as they stress the need to reach out to a young adult population that can still be vaccinated. Also, our work provides researchers a method to better use social media or large amounts of textual information to understand and communicate based on how a certain population represents domain knowledge of a health topic.

## Appendix Table A

**Table A1.** Top 10 Nearest Neighboring Concepts (Reddit Corpus).

big_pharma 0.467084: profit 0.460571: vaccines 0.458160: make 0.456284: research 0.456098: vaccine 0.451586: extremely_poor 0.448948: companies 0.442906: people 0.438898: profit_greatly 0.438278: developed	cancer 0.845071: cervical_cancer 0.798211: hpv 0.782156: cancers 0.766900: lead 0.747220: causing 0.742168: caused_by 0.724137: developing 0.714363: genital_warts 0.711839: related 0.708041: infection	death 0.547633: reported 0.530533: severe 0.522110: vaccine 0.522073: diseases 0.519841: caused 0.517385: meningitis 0.516819: important 0.509423: children 0.508800: deaths 0.508455: cervical_cancer
doctor 0.813682: told 0.778519: told_me 0.772079: doctors 0.770212: time 0.769764: checked 0.761214: find 0.761100: thought 0.752800: talk 0.750967: gynecologist 0.750645: thing	family 0.697475: things 0.695137: talk 0.686675: friends 0.683518: good 0.679167: feel 0.675407: thing 0.671799: doctor 0.657793: hard 0.656534: life 0.655129: time	genital_warts 0.833893: strains 0.822480: warts 0.802735: strain 0.792468: hpv 0.753186: cervical_cancer 0.736774: types 0.726422:90 0.714363: cancer 0.692250: visible_warts 0.683885: caused_by
health 0.722293: find 0.717309: sexual 0.705585: reason 0.705398: means 0.704923: current 0.703309: thing 0.701957: people 0.698969: past 0.696766: as_well 0.695444: lot	hpv 0.840775: cervical_cancer 0.820574: infection 0.803351: common 0.798211: cancer 0.797479: strain 0.796438: means 0.796064: symptoms 0.792468: genital_warts 0.790667: specific 0.788417: women	hpv_vaccine 0.681349: wiki 0.650689: herpes_simplex_virus_2 0.613281: human_papill 0.575218: epidemiology_of_herpes_simple 0.562787: human_papillomavi 0.557843: human_papillomavir 0.544148: cite_note 0.539670: epidermodysplasia_verruciform 0.532161: the_immortal_life_of_henriett 0.514284: vaginal_cancer
inaccessible 0.388583: nucleotides 0.324041:12,000_cases 0.303700: removal 0.298032: quarter 0.297910: premature_ovarian 0.294843: harms 0.287773: scc 0.285973: urologist 0.284799: aposto 0.283750: advancements	information 0.618781: thing 0.617080: read 0.613979: understand 0.611145: absolutely 0.600797: find 0.599728: literally 0.599621: point 0.596949: time 0.595125: people 0.592996: make	myth 0.391901: present 0.387535: stds 0.383372: claims 0.382261: hygiene 0.378389: disease 0.374891: sexual 0.371264: provided 0.371161: passed 0.368088: easily 0.366686: actual
pain 0.687804: painful 0.684260: hurt 0.652976: feeling 0.608038: bleeding 0.580629: bad 0.579333: discomfort 0.558158: felt 0.555020: hurts 0.553561: cramping 0.552674: feel	prevention 0.569931: hiv 0.547458: risk 0.542518: circumcision 0.538610: hiv_transmission 0.535975: prevent 0.530347: infection 0.530346: significant 0.528600: reduces 0.519289: sexually_transmitted 0.514680: infections	promiscuity 0.554718: people 0.548820: agree 0.541454: issue 0.535961: fact 0.530874: thing 0.529724: real 0.526339: problem 0.523906: idea 0.523368: reason 0.522908: problems
recommendation 0.755715: immunized 0.737165: favor 0.652360: infants 0.645749: leads 0.629266: this_association 0.529488:_198 0.506701: if_suitable 0.442810: thimerosal_containing 0.417413:_johnson 0.395895: alternative_preparations	risk 0.802331: chance 0.801576: infection 0.775372: transmission 0.773495: hiv 0.760732: hpv 0.759224: contracting 0.753542: low 0.747165: transmitting 0.741573: risks 0.741290: chances	scientific_evidence 0.602846: demonstrates_potential 0.599960:_existing 0.480253: medical_benefits 0.432091: circumcision 0.409245: policy.pdf 0.393124: evidence 0.390793: sufficient 0.364787: argue 0.361523: reducing 0.357875: benefits
side_effects 0.626167: taking 0.556852: gardasil 0.556289: vaccine 0.554174: effects 0.553798: vaccines 0.522590: shot 0.521854: severe 0.519835: caused 0.510788: long_term 0.510151: safety	trust 0.763864: things 0.748168: good 0.744503: relationship 0.738419: thing 0.734813: honest 0.734801: time 0.731637: past 0.726523: find 0.724313: lot 0.719553: situation	unnecessary 0.613867: worth 0.605971: vaccinations 0.595852: reason 0.568391: fact 0.567142: people 0.558949: basically 0.558437: lot 0.548310: due 0.544150: point 0.544112: health
unsafe 0.633610: sex 0.576699: safe 0.519984: gay 0.517893: having_unprotected 0.513171: practice_safe 0.511257: safer 0.510768: encourage 0.510246: unprotected_oral 0.506795: avoid 0.505143: partners		
